# Improving the quality of long-term care services in workforce dimension: expert views from Australia and South Korea

**DOI:** 10.1186/s13690-022-00872-9

**Published:** 2022-04-07

**Authors:** Hyo Young Lee, Stephanie Short, Mi-Joung Lee, Yun-Hee Jeon, Eunok Park, Young-Ran Chin

**Affiliations:** 1 Department of Health Administration, Dongseo University, Busan, Republic of Korea; 2 Sydney School of Health Sciences, The University of Sydney, Sydney, Australia; 3 School of Nursing and Midwifery, The University of Sydney, Sydney, Australia; 4 College of Nursing, Jeju National University, Jeju-si, Republic of Korea; 5 Department of Nursing, Chungwoon University, Chungnam, Republic of Korea

**Keywords:** Workforce, Quality, Long-term care, Competence

## Abstract

**Background:**

The long-term care workforce is an essential factor in the provision of qualified long-term care services. Identifying workforce issues can help developing countries in East Asia and the Pacific prepare for the increase in the older population. Their experiences can be used as lessons for other countries. This study aimed to identify the workforce issues that should be addressed in order to provide high-quality long-term care services for older adults.

**Methods:**

In-depth interviews and content analysis were conducted with a purposive sample of long-term care experts. There were eight participants from Australia and 14 from South Korea. The participants were questioned on important workforce issues to improve the quality of long-term care services. These were open-ended questions that comprised ideas derived from the literature. Major themes were systematically and comprehensively classified and coded to examine recurring comments and themes.

**Results:**

The issues in the two countries were very similar: labor shortages, inadequate working conditions, insufficient career and staff training, and the need of counselors or consultants for finding proper services. There were also differences in terms of competency of the service operators and their corresponding multicultural competency.

**Conclusions:**

Providing high-quality long-term care service requires multipronged approaches to workforce capacity and work environment. An adequate and competent workforce should be established to match the service needs of the older population. To improve quality, better working conditions and improved motivation to work in care for older people should be considered. Concurrently, each country would need a workforce strategy tailored to different conditions and environments. This should include policies to induce an influx into the workforce.

## Background

Australia and South Korea are both members of the Organisation for Economic Co-operation and Development (OECD) in the East Asia and Pacific region. These two countries established diplomatic relations in 1962 and, under a free trade agreement from 2014, have continued to more actively promote exchanges [1, 2]. They both have made efforts to make universal health care available to the public [3]. Common to both countries is also their social structure and certain social phenomena, such as population age structure and extended life expectancy [2]. The OECD countries and countries that lead in long-term care (LTC) services have been making efforts to provide appropriate and sustainable LTC services [4]. In Australia, LTC services are mainly provided by non-governmental providers and funded by the Australian government, but also supplemented with clients’ out-of-pocket expenses. Such services in Korea are delivered under social insurance with insurance fees [5, 6]. The proportion of the older population in both countries is growing at similar rates—from 13.6% in 2010 to 16.3% in 2020 in Australia and from 10.8 to 15.7% in South Korea [7]. With this, both countries have tried to maintain the quality of LTC care by reforming and developing support systems [5, 6] since they need more workers to care for older people than ever before. Australia launched LTC system in 1997 [8], even before South Korea, which launched its system in 2008 [5].

Health and medical resources, including health and medical personnel, are basic elements indispensable to the supply of health care [9]. Further, the workforce is one of core components for the quality of care [4, 10–12]. If the staff and care workers are inadequate or underqualified, then quality is not guaranteed. Among workforce issues in the areas of LTC, challenges in human resource such as turnover and wages are important factors associated with the quality of care [13]. There are also reportedly statistically significant differences in staffing hours per resident day for patient-related hours [14]. In the U.S., the development of a sufficient and qualified professional LTC workforce was found to depend on the national ability to increase and improve the effectiveness, efficiency, and availability of training [15]. As we are facing an unprecedented global demand for LTC, it is important to clarify the need for care workers who are qualified to provide such care. The availability these workers must also be ensured.

Many issues have been raised regarding LTC workers; these include the lack of suitably skilled and qualified staff to provide services [2, 16], importance of respectful attitudes toward older individuals [17], communication competency of care staff [18], and availability and quality of training programs for care workers [19]. Preliminary research suggests that a major possible reason for inadequate care in Australia was staff shortages owing to the demands arising from fewer skilled staff present to meet clients’ needs. As such, the shortage in staff has been identified as a possible key cause of insufficient residential care [16]. Reports from one type of LTC facility suggest care workers are overloaded with tasks, and therefore engage sparingly with residents while addressing the latter’s problems [20, 21]. In Canada, a positive attitude by the nursing staff toward older people is considered an important factor in the quality of care provided in LTC facilities [18]. In addition, cross-cultural care and communication are needed when staffing LTC facilities. This is because a linguistically diverse staff adds value and improves the quality of service [19].

South Korea and Australia lead LTC in the East Asia and Pacific region, especially in their preparation for an aging population [4]. The aim of this study is to examine the workforce issues that must be addressed to provide high-quality LTC services for older individuals, especially from the perspectives of the common issues and differences between the two countries. The expert perspectives of workforce issues present in these two uniquely positioned countries will provide insights to improve the quality of LTC services in both countries.

## Methods

### Study design and participants

One-on-one in-depth interviews and content analysis were used, focused on direct care workers, in order to provide insights for improving the quality of LTC services in both countries. For this purpose, we selected participants who have been actively engaging in LTC for 10 years or more. We collected qualitative data with eight Australian and 14 Korean LTC experts through two to four-hour interviews. Between January and August of 2019, purposive sampling was used to find long-term care experts via LinkedIn, Google Scholar, the registry of LTC experts by the Korean Health Insurance Corporation, literature reviews, and Internet web searching for “aged care,” “long-term care,” “long-term care (aged care) workforce (staff, experts, personnel),” or “service for the older adults.” Such sampling ensured that we collected the perspectives of academic experts (researchers or professors), service providers (senior staff), and the staff of governmental organizations. We requested 20 individuals from each country to participate; 8 from Australia and 14 from Korea agreed to participate. Potential interview participants were contacted via e-mail to provide information about the interview; written informed consent was obtained from the participants before interviews were scheduled. Verbal consent was also recorded in their workplaces before starting the interview (Table 1).

**Table 1:** Characteristics of the participants unit: n

	Australian (*n* = 8)	South Korean (*n* = 14)
Sex		
Male	–	5
Female	8	9
Age (years)		
30–39	2	3
40–49	2	4
50–59	3	6
60–69	1	1
Affiliation		
Academia	4	4
Quality control organizations	2	1
Service provider	2	9

### Interview questions and process

After intensive literature reviews and meetings among the researchers, the basic interview questions were created, and responses to the basic questions led to detailed follow-up questions meant to explore the major workforce issues (focusing on the care givers) to improve the quality of the service. The interview questions were developed by six experts with doctoral degrees or higher. They were PhDs in nursing or public health who had extensive experience in conducting qualitative interviews in the field of LTC. The main interview questions were as follows: “What do you think is the most ideal way to operate for providing high-qualified LTC services?”, “To improve the quality of LTC service, which aspect of LTC services should be improved?” Once the participants answered these questions, we asked them to explain their rationale for each question. We then asked more questions in relation to workforce issues, such as “In the part suggested above that supplementation is necessary for quality service, what are the important workforce issues that should be addressed to improve the quality of LTC services?” and “In the LTC area, are there any concerns related to care workers for providing good services?” Interviewers also asked for more feedback before asking a secondary question by adding questions related to service personnel: For example, “What do you think are the most important competencies (skills) of service-providing personnel in long-term care?” The respondents were then asked to state their reason for each response as well. Interviews were conducted until there were no new concepts or issues found regarding the LTC workforce.

### Analysis and ethical considerations

The interviews proceeded for two to 4 h. All interviews were transcribed, and themes of workforce issues were extracted using content analysis. The major themes from the interviews were then systematically and comprehensively classified and coded to examine recurring comments and themes. This was done through a simple counting of sections of text that contained similar words and phrases. This research was approved by the Human Ethics Committee (1041493–201,711-HR-008-01). Additionally, the six researchers held degrees of Doctors of Philosophy in Nursing or Public Health and had extensive experience in conducting qualitative interviews or in the field of LTC. Among them, three interviewers were all registered nurses and two were physiotherapists.

## Results

### Common workforce issues in Australia and South Korea

**Fig. 1:**
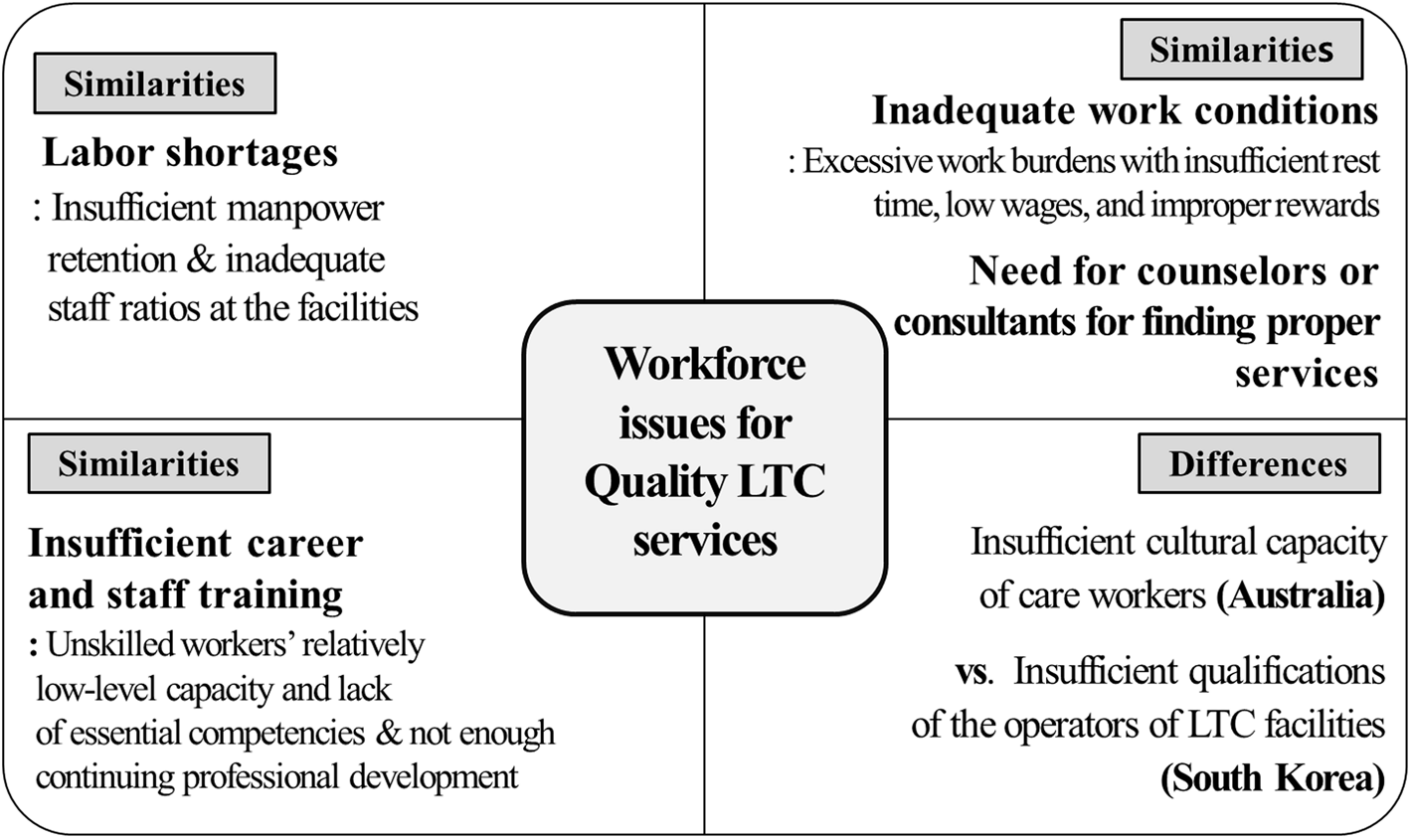
LTC workforce issues between Australia and South Korea

Regardless of the launching year of LTC services, the workforce issues directly involved in providing services to older individuals in Australia and South Korea had much in common. The four main issues that appeared in common were labor shortages, inadequate working conditions, insufficient career and staff training, and need of counselors or consultants for finding proper services (Table 2). All of these issues may be influenced by each other because the raised concerns are closely linked. Figure 1.

**Table 2:** Common workforce issues relating to the improvement of the quality of LTC services in Australia and South Korea

Themes/ sub-themes	Quotes
**1. Labor shortages**: Insufficient manpower retention & inadequate staff ratios at the facilities	• The lack of registered nurses (RNs) was a serious problem in the residential facilities (Participant B in Australia). • The number of care workers may decrease once the accredited staffs are gone. The staff members are mostly casual workers (Participant G in Australia). • There are difficulties conducting a cognitive and physical activity program due to labor shortages (Participant A_1 in South Korea). • The lack of nurses and nursing services in the LTC program are issues. The quality of LTC will improve when we secure the proper number of nurses who can work in LTC facilities (Participant J_1 in South Korea). • The staffing ratios of the day-care service should be realistic. The ratio should be 1 (career) to 5 (staff members) for LTC service. The current ratio is 1 (career) to 7 (staff members), and that operation cannot provide a variety of services to clients (Participant D_1 in South Korea).
**2. Inadequate work conditions**: Excessive work burdens with insufficient rest time, low wages, and improper rewards	• It is necessary to improve the environment so that staff can work effectively with sufficient rest time (Participant C in Australia). • The wages in the field of LTC are too low for RNs. The same level of pay is necessary for RNs working in LTC as for those working in a hospital (Participant F in Australia). • They would have pride in their job if working conditions were improved and wages were increased by adjusting governmental subsidies (Participant G_1 in South Korea). • Improving working conditions is an urgent matter, as workers often leave their jobs within 1 to 2 years. We need to show a clear career path for caregivers by increasing wages – to improve the quality of candidates (Participant N_1 in South Korea). • Improvement is needed in relation to the collective burden of direct service staff. This problem occurs when they record information on the system according to the evaluation standards (Participant A_1 in South Korea). • The evaluation items are too broad and complicated in a situation with poor working conditions (Participant D_1 in South Korea).
**3. Insufficient career and staff training:** Unskilled workers’ relatively low-level capacity and lack of essential competencies & not enough continuing professional development	• The capacity of the workforce is lower than the required level. The most important competency of care workers is the ability to understand the social situation and the health of the consumers (Participant A in Australia). • In particular, it is very important to care for dementia cases. The ability of the workforce to manage diabetes is also critical. Care workers need basic LTC skills, which require lifting clients, communication skills, and personal care skills. It is also very important to form good relationships with the clients. There is a huge gap between care workers and competencies (Participant E in Australia). • Care workers need communication skills to communicate with people about their care needs and their emotional and social problems. Client-centered care competency is required in a care worker. They must have the ability to identify the clients’ emotional and social needs and [they must be able to] build a care plan. However, unskilled care workers are [also] a frequent issue (Participant C in Australia). • Continuing professional development for care workers is necessary; including manual handling, fire safety training, and infection training. Palliative care and training for dementia are required. Continuing professional development is also essential to improve the quality of the services (Participant F in Australia). • Many caregivers do not have the ability to provide services even if they have sufficient practical training in the field because they have a poor academic background (Participant H_1 in South Korea). • Education for caregivers, including work ethics, should be strengthened (Participant B_1 in South Korea). • Continuous training for caregivers should be more systematically improved (Participant D_1 in South Korea).
**4. Need for counselors or consultants for finding proper services**	• I think the information technology system is very useful for obtaining data on older people, but it is inconvenient for older individuals themselves to use and should be supported by the workforce (Participant A in Australia). • We need a person who can help us understand the system and [who could] choose services within our budgets [for us] (Participant B in Australia). • We need someone like a service navigator to help people use the service. The service clients need help from someone because online information is not sufficient to solve all the problems involved in getting help or advice (Participant C in Australia). • Case managers provide advice, make plans, and check the changes. Therefore, their roles are very important. This should be a public service; but presently, it is a service provided by the providers (Participant D in Australia). • Currently, no one is taking on the role of the care manager at present. There are many gaps in the service, and [many] parts that need to be revised (Participant B_1 in Korea).

#### Labor shortage: insufficient manpower retention, and the low level of staff ratios

Participants raised the issue of insufficient manpower retention and the low level of staff ratios for customers caused by insufficient numbers of care workers. In particular, participants stressed the lack of registered nurses; however, this seemed to be a challenge for the local community, as it is difficult to recruit nurses in the LTC sector (*Participant G in Australia*). Participants also noted that the legally required staff ratios should be increased through policy efforts if it cannot be improved voluntarily by service providers. The number of direct care personnel required to work in the facility has not been met (*Participant A in Australia*), and, to compensate for the lack of staff, work shifts are overlapped (*Participant B in Australia*), which could be the cause of the high work burden. Participants emphasized that the system must reduce administrative tasks, and they needed government support to provide sufficient services for those who are eligible for the LTC services (Table 2).

#### Inadequate work conditions

With regard to working conditions, participants reported excess work burdens with insufficient rest time (*Participant C in Australia, Participant A_1 in South Korea*), low wages (*Participant F in Australia, Participant G_1 in South Korea*), and improper rewards (*Participant F in Australia, Participant N_1 in South Korea*), which can induce care workers to leave their jobs. Insufficient rest time could result from labor shortages. Thus, working conditions need to improve, which will allow workers to serve longer. The issue is described in detail in Table 2. Traditionally, care workers’ jobs in the two countries were low-income positions. We believe that a lack of proper working conditions to cope with hard physical labor will eventually lead to a negative circulation structure that cannot guarantee the sustainability of jobs.

#### Insufficient career and staff training

In the theme of “insufficient career and staff training,” “unskilled workers’ relatively low-level capacity and lack of essential competencies,” (*Participant A, E in Australia, Participant H_1 in South Korea)* and “not enough continuing professional development” *(Participant C in Australia, Participant D_1 in South Korea)* were frequently reported. Even though care workers received mandatory education, they need better ability to understand clients, communication skills, work ethics, and practical training in the field. This could also be associated with the low sustainability of their jobs. Participants also noted that mandatory education and continuing professional development should be intensified through policy and voluntary service providers (Table 2).

#### Need for counselors or consultants for finding proper services

There is a lack of personnel for essential roles, such as counselors, consultants, and case managers, for finding high-quality and proper support for clients (*Participant A, B, C in Australia, Participant B_1 in South Korea)*. The participants strongly reported a “need for a person who counsels consumers and gives advice and information to consumers” *(Participant A, B in Australia)* and a “need for case managers and care planners (*Participant B_1 in South Korea)*.” Consumers need a person who can counsel clients and provide information on choosing a good provider to provide for LTC services within their budgets. Furthermore, this person can assist them in formulating a care plan. All of reports from participants were related to access of the services and the desire to receive high-quality LTC services.

### Different workforce issues relating to insufficient career development and staff training

There is a need for multicultural competence among Australian care workers because of the cultural differences and the non-English backgrounds of both clients and workers. In South Korea, however, proper management, mindset, efforts, and passion by operators were identified as important factors, which need to be strictly verified before sanction is sought for establishing a LTC facility. Both issues can be included in “insufficient career and staff training,” but a different competency or importance of a specific person was mentioned; therefore, we classified it as a difference. Australia is a multicultural society with a great number of migrant workers and clients. Thus, the lack of multilingual and cultural competencies might be a cause for concern [22]. Language interpretation and understanding cultural differences are thus part of necessary competency for Australian care workers (Table 3).

**Table 3:** Different workforce issues relating to insufficient career development and staff training

		Differences (Australia vs. South Korea)	
		Themes	Quotes
**3. Insufficient career development and staff training**	Australia	**Insufficient cultural capacity of care workers in Australia**	• Some multilingual staff are required in LTC settings. There are many immigrants, so there is a problem with language interpretation (Participant A in Australia). • There is a need to consider using language pictures, as the clients have a different perspective on what they want. However, this is not simply a matter of English; it is a cultural difference (Participant C in Australia). • The cultural and linguistic issue is part of the certification assessment; thus, we should be continually trying to fill the needs of consumers with multicultural backgrounds (Participant F in Australia).
	South Korea	**Insufficient qualifications of the operators of LTC facilities in South Korea**	• Proper management on the part of the operator of a facility is the most important factor for improving service quality (Participant E_1 in South Korea). • The mindset of the operator is very important as it impacts employees (Participant K_1 in South Korea). • Strengthening the requirements of the operator should be considered. The efforts of the operator, combined with a passion for their work on the part of the care workers will create a qualified LTC service. Competence and effort are the best traits for operators. I think that the service manager should endeavor to take complete responsibility for providing services effectively and conscientiously (Participant H_1_in South Korea).

## Discussion

Our study describes workforce issues that are common to South Korea and Australia, two countries that lead LTC services in the East Asia and Pacific region despite have different timelines. The study posits that their experiences can provide mutual insights as well as insights for other countries seeking to improve the quality of LTC. We believe policies should address the “active enlargement of the LTC workforce”; they should focus on “strengthening basic education and continuing professional development,” “reinforcing the roles of each professional or need and easy access to a person for finding proper services,” “improving working conditions and better treatment of service workers,” and ensuring a supportive system that includes financial expansion to supplement care workers.

Our survey results show that both countries have been struggling with workforce shortages. Previous studies reveal that nursing homes are already short-staffed, and care workers are overworked and can rarely engage with residents [21, 23, 24]. While the Korean government has set official staff ratios [23], Australia has not, but the Australian Nursing and Midwifery Association recommends that minimum care requirements should include 30% registered nurses, 20% enrolled nurses, and 50% personal-care assistants [25, 26]. Both countries still face difficulties with worker retention in LTC facilities because workers frequently leave owing to low wages and poor job satisfaction [26]. While the importance of nurse staffing ratios has been emphasized, this still varies among residential facilities [27]. The staffing ratios should be realistic and strengthened to enhance the quality of care. Ensuring a skilled workforce should be the most critical aspect of the quality assurance measures in the care of older people [28]. In addition, efforts to enhance the positive image of caregivers are needed because of the negative connotations of the job—few young and qualified workers want to enter this field. It is thus important to change perceptions of the nature of this work to that of a specialized job in the field of care work.

OECD countries use informal care workers to address the shortage of manpower, accounting for up to 20% of the workforce. Belgium, Austria, the Czech Republic, the United Kingdom, and Germany accounted for nearly 20%, while Australia had 14% in 2018. LTC is a labor-intensive field, and effective and efficient use of manpower is required for the continuity of LTC services [29]. Two thirds of OECD countries provide cash benefits to family caregivers or periods of paid leave for their informal caregivers.

The measure introduced to reduce the demand for LTC services and manpower in South Korea is family care [30]. In other words, if one of the family members obtains a professional caregiver qualification and provides services to a family member for more than 60 or 90 min a day, they are eligible to receive about $363 USD or about $758 USD from the National Health Insurance Corporation, respectively. In addition, to take care of a family member with mild dementia, they must receive additional dementia specialized education. Although there is no study that proves how much family care has reduced the demand for caregivers, it is judged that the possibility of family care can be further expanded in the future as there are still many individuals who want to obtain a caregiver qualification. As a way to compensate for the lack of workforce, part-time workers who want to increase their hours, unemployed individuals, and inactive former informal caregivers may be recruited into the LTC workforce. In line with this, workers who want to delay their retirement and young students should also seek ways to increase manpower. It is fundamentally essential to create sustainable working conditions, and if the work environment improves, the influx of men into the LTC workforce will also increase [31].

Australia offers various careers in the LTC field; among such careers [32], the proportion of nurses is higher in Australia than in Korea [5]. Additionally, volunteers in Australia in LTC settings form about 9% of the staff in residential care and 25% in community care. The lack of a professional workforce is not a simple problem. Caregivers should generally be well paid in order to attract workers in this field of work. In Australia, present wage levels for health care assistants are low; the growing number of migrants has also exacerbated the effect of lack of multilingual and cultural competencies in care workers [22]. In South Korea, a lack of cultural competency is not an issue, as immigrants comprise only 4.21% of the population [33]. However, South Korea should consider the possibility of the numbers of immigrants increasing in the future [5]. In South Korea, holders of residence visa (F-2); overseas Koreans (F-4); and holders of permanent resident- (F-5), marriage immigrant- (F-6), and visiting employment (H-2) visas are allowed to obtain caregiver qualifications. Hence, the proportion of foreign caregivers is expected to increase in the future, and multicultural and communication skills of caregivers are expected to become important, such as in Australia [34]. The Australian Aged Care Quality Agency reports that, among LTC facilities that did not meet quality standards, the problems were most often related to human-resources management [35].

Essential competencies should be strengthened by entry-course training and continuous professional development for staff and care workers through active support of the operators and providers. Essential competencies that were mentioned during interviews included personal-care skills, communication skills, safety and food management, infection control, manual handling, dementia care, and diabetes management. The curriculum should be managed to improve these skills and competences, as these are essential for care workers. There is a critical shortage of appropriately skilled and knowledgeable staff in LTC services in Australia, particularly registered nurses [36]. The interviewees and a previous study also report that one of the competencies required in LTC settings is that of dementia care [37]. The disciplines of gerontology and geriatrics should be also strengthened within the practices of the LTC environment [37]. In Korea, an additional insurance benefit is given to facilities to induce sufficient quality manpower such as registered nurses [31]. The training hours for LTC caregivers vary from country to country. In the United States, direct care workers receive 75 h of training, while in Japan, certified care workers receive 1800 h of training and early career care workers receive 130 h of training [38].. In addition, in Korea, 240 h of education is mandated and strictly managed as per national qualifications [31]. In Australia, by granting accreditation to educational institutions, each educational institution operates a different curriculum, encouraging each such institution to voluntarily improve the quality of education [5].

In Korea, continuing professional development for care workers is offered, which includes a special dementia care course. This program runs for 60 h, and care workers are required to pass an exam to complete the course but depending on their type of qualifications, the training hours will vary slightly. This is not a mandatory program, but workers receive incentives for responding to the provision of dementia care [39]. In Australia, manual handling of patients, fire-safety training, and infection control training are all mandatory for care workers [28]. Greater awareness of skills and practices, as well as the development of confidence in working with complex situations, could enhance the role of care workers and the quality of service [20]. Among LTC care workers in OECD countries, approximately 17% of the workers are on temporary employment, and this reduces the opportunity for strengthening their capacity, does not guarantee job continuity, and involves low pay [29]. Except for nurses, social workers, and therapists, their wages are below the national average; thus, it is urgently necessary to address this concern. Further, these workers experience more adverse social behaviors such as verbal abuse and physical violence while still having a closer relationship with their patients, as compared to other workers in the health care field [30]. South Korea only stipulates the qualification standards for instructors in caregiver education [31], but there are no qualification requirements for the head of an institution, who determines the overall direction of educational institution operation. Thus, the chairperson’s qualification management plan should be discussed in the future studies.

Other discussion points involve the need for a supportive workforce for accessing LTC information that could compensate for lack of competency of operators. In 2015, Australia moved the process of applying for LTC online [40]. The website for the LTC service called “My Aged Care” has been tested to provide easy access to information, including help from a LTC advocate through the *Older Persons Advocacy Network* [41]. However, Australian experts report that older consumers were uncomfortable with online access; thus, some consumers may not be getting enough information, advice, or counseling. Therefore, supportive workforce should still complement online access. With regard to the importance of operators’ competency, evidence shows that leadership is critical for achieving good health outcomes for residents, and good quality care may cost no more than poor quality care [12, 42]. Among the greatest challenges to the capacity of LTC service is, first, delivering high-quality services to the current older population, and, second, the expected inadequacy of our workforce to deal with future aging. This shall include both the numbers of workers and the quality of their training [4]. Policy efforts and employment policies should both work to secure the right characteristics and types of personnel for long-term care services and to improve the quality of employment in the LTC services sector.

It is notable that two different structures on which the LTC was built (non-governmental providers and funded by government, but also supplemented with clients’ out-of-pocket expenses, subsidized versus social insurance with insurance fees) are facing similar problems. By sharing their respective experiences in the supply of efficient health care services, both countries can gain the expertise and wisdom needed to develop more advanced health care services. Therefore, the LTC experience in each country may indicate the improvements needed in both countries’ services. The two countries’ experience with LTC workforce issues can offer lessons to other countries as well. The factors discussed herein affect the older population’s life. Of these, the most critical are *labor shortages*, *inadequate working conditions*, and *insufficient career and staff training*. Fixing these issues will help extend necessary LTC to more populations and incentivize providers to improve their services as well. For example, older populations and their family have expressed “the need for counselors or consultants for finding proper services,” especially more customer-friendly services through system improvement.

## Conclusion

Workforce concerns in South Korea and Australia are similar. An adequate number of care workers and a satisfactory degree of competency are needed if the workforce is to provide high-quality services. To improve service quality, fair salaries, efforts to improve social awareness, and encouragement to enter LTC work should be considered. This should also include policies that attract a skilled workforce. Organizations should also create a culturally sensitive trained workforce and establish competency requirements for operators. Continuously identifying and supporting issues related to providing services from the perspectives of the providers, care workers, and consumers should follow. Furthermore, solutions must be developed for workforce issues in order to provide high-quality and sustainable services to the older population.

Nevertheless, our study has several limitations that should be addressed in future studies. First, it is crucial to expand the sample size to various sources. We only interviewed a small number of LTC experts. Second, this study focused on the overall aspects of important workforce issues that need to be considered to provide high-quality LTC services. There exist more specific issues related to each theme that may be identified in future studies. The issues of all specialized care workers were not identified in detail, even though personal-care workers were the main employees. Having said that, this study found that South Korea and Australia experience similar workforce issues, but also suffer from issues unique to each country. The growing number of LTC services and the issue of providing and managing people to provide quality services are common to many countries. Hence, the insights from the current study can be useful in assessing the LTC status of other regions.

## Data Availability

The datasets are available from the corresponding author through the meeting of the Ethics Committee.

## Abbreviations

OECD: Organisation for Economic Co-operation and Development
RN: Registered nurse
LTC: Long-term care
